# Supporting Breastfeeding in Early Childhood Education and Care Settings in Italy: A Relational and Cultural Analysis of Maternal and Educator Perspectives

**DOI:** 10.3390/children13020227

**Published:** 2026-02-05

**Authors:** Angelica Arace, Protima Agostini

**Affiliations:** Department of Philosophy and Education Sciences, University of Turin, 10124 Turin, Italy; protima.agostini@unito.it

**Keywords:** breastfeeding, early childhood education and care (ECEC), childcare services, maternal well-being, parental ethnotheories, educator perspectives, continuity of care, family engagement, intensive motherhood

## Abstract

**Highlights:**

**What are the main findings?**

- Maternal beliefs about breastfeeding and the willingness to continue breastfeeding within early childhood education and care (ECEC) settings show a strong association with parental ethnotheories that privilege relational closeness, interdependence, and continuity of care.
- Educators view breastfeeding in childcare as a relationally meaningful practice, particularly in terms of parental engagement and educational continuity. They see it as supporting both the child’s emotional security and maternal well-being, while placing only minimal demands on organizational routines.

**What are the implications of the main findings?**

- ECEC services can act as pivotal relational environments that either facilitate or constrain the continuation of breastfeeding beyond the home, depending on the organizational climate and the degree of support provided by educators.
- Breastfeeding support practices within ECEC should extend beyond logistical accommodations. They call for relational, non-prescriptive approaches that acknowledge the cultural plurality of caregiving beliefs and actively counter the adverse effects associated with “intensive motherhood.

**Abstract:**

**Background/Objectives**: Breastfeeding is widely acknowledged for its benefits to child development and maternal well-being. Yet breastfeeding practices often decline during early childhood transitions, particularly when children enter early childhood education and care (ECEC) services. Research has largely concentrated on healthcare contexts, leaving the educational domain comparatively underexplored in its potential to sustain or hinder breastfeeding continuation. This cross-sectional study examines associations between maternal beliefs regarding the value of breastfeeding and personal, relational, and contextual factors related to breastfeeding continuation within ECEC settings. It also incorporates educators’ perspectives and situates breastfeeding practices within the broader framework of parental ethnotheories. **Methods**: The study formed part of a pilot parental engagement initiative involving 17 childcare centers in Northern Italy, designed to promote dialogue and supportive practices around breastfeeding within ECEC services. This context is acknowledged when interpreting findings related to organizational climate and educator perspectives. Participants included 490 mothers of children enrolled in ECEC services and 118 educators. Mothers completed questionnaires assessing breastfeeding beliefs and experiences, co-sleeping practices, perceived social support, and parental ethnotheories (independence versus interdependence). Educators reported on their experiences in supporting breastfeeding within the childcare context. Analyses included descriptive statistics, analyses of variance, chi-square tests, and binary logistic regression to examine factors associated with breastfeeding continuation. **Results**: Mothers strongly endorsed the developmental benefits of breastfeeding and expressed greater alignment with caregiving practices emphasizing interdependence and physical proximity. Continued breastfeeding within childcare settings correlated with interdependence-oriented ethnotheories, younger child age, and higher engagement in co-sleeping practices. Educators reported generally positive views of breastfeeding in childcare, highlighting its contribution to children’s emotional security and maternal calm, alongside its negligible impact on educational organization. **Conclusions**: ECEC services play a crucial role in sustaining breastfeeding beyond the domestic sphere. Relational practices and organizational climates that welcome breastfeeding can foster continuity of care, strengthen parental engagement, and promote child well-being.

## 1. Introduction

Breastfeeding is widely recognized as a key caregiving practice with well-documented benefits for infant health, development, and maternal well-being [1,2,3,4,5]. International guidelines recommend exclusive breastfeeding during the first months of life and its continuation alongside complementary feeding thereafter [6]. Despite these recommendations, breastfeeding practices often decline over time, particularly during early childhood transitions such as the return to work and the child’s entry into early childhood education and care (ECEC) services, highlighting the persistence of structural, cultural, and organizational barriers [7,8,9].

Research on breastfeeding has traditionally been situated within biomedical and public health frameworks, focusing on maternal knowledge, clinical support, and health outcomes [3,10]. While this literature has provided robust evidence on the benefits of breastfeeding and the effectiveness of healthcare-based interventions, it has also highlighted important limitations. In particular, breastfeeding support interventions tend to be most effective in the early postnatal period and less successful in sustaining breastfeeding over time, especially once families move beyond healthcare settings into everyday caregiving environments [11].

In recent years, an expanding body of research has emphasized the psychosocial and emotional dimensions of breastfeeding [12,13]. Qualitative and mixed-methods studies have shown that breastfeeding is experienced not only as a feeding practice but also as a deeply relational and emotionally charged experience. Breastfeeding difficulties, perceived pressure, and moralized expectations surrounding “good motherhood” have been consistently associated with maternal stress, anxiety, and ambivalence [14,15,16]. Systematic reviews and meta-analyses further demonstrate that emotions and injunctive norms—social expectations about what mothers ought to do—play a significant role in shaping breastfeeding intentions, experiences, and continuation [17].

These findings challenge normative approaches to breastfeeding promotion that prioritize duration and exclusivity without sufficient attention to maternal well-being and lived experience. Rather than reflecting individual inconsistency or lack of commitment, maternal ambivalence around breastfeeding appears to be a structurally produced response to competing cultural expectations, intensive mothering ideologies, and everyday caregiving demands [15,17,18].

From a cultural and developmental perspective, breastfeeding practices are closely linked to broader parental ethnotheories—culturally embedded belief systems that guide caregiving goals and practices in early childhood [19,20,21].

Parental ethnotheories constitute culturally shared systems of beliefs, values, and expectations that shape not only parents’ developmental goals for the child, but also the everyday caregiving practices through which these goals are pursued. As emphasized by Keller [19], ethnotheories do not operate solely at a declarative or ideological level; rather, they are translated into coherent patterns of parental behavior that organize how the child’s body is cared for, regulated, and embedded in social interaction. From this perspective, practices such as breastfeeding and co-sleeping should not be understood as isolated or contingent choices, but as concrete expressions of broader cultural orientations to caregiving.

In particular, ethnotheories oriented toward interdependence and relational proximity tend to value practices that maintain bodily and emotional continuity between caregiver and child, including prolonged breastfeeding, shared sleep arrangements, and other forms of caregiver-guided regulation of infant needs. These practices support immediate responsiveness to the child’s signals and shared emotional regulation, consistent with a view of development as an inherently relational process. By contrast, ethnotheories more strongly oriented toward early independence tend to prioritize practices that promote physical separation, self-regulation, and autonomous exploration, rendering prolonged breastfeeding and nighttime proximity less central.

The link between ethnotheories and specific caregiving practices is therefore theoretically grounded insofar as ethnotheories provide an interpretive framework that assigns meaning to caregiving practices and shapes their cultural legitimacy. In this sense, the co-occurrence of practices such as co-sleeping and breastfeeding within the same parental profile does not merely reflect behavioral association, but rather indicates the presence of a coherent system of beliefs that structures caregiving experiences in early childhood.

These orientations are not merely individual preferences but part of coherent cultural models of care that shape how parents interpret children’s needs and their own parental roles [19].

Breastfeeding is therefore increasingly conceptualized as a relational, cultural, and organizational practice shaped by parental ethnotheories and institutional contexts [19,22]. Cultural models of parenting influence how breastfeeding is interpreted as a moral obligation or marker of good motherhood, often embedding it within ideologies of intensive mothering [17,18]. The concept of intensive mothering, as formulated by Sharon Hays [17], describes a cultural model of parenting that assigns mothers a primary and continuous responsibility for their child’s well-being and development. Within this model, motherhood is conceived as an activity requiring a constant investment of time, emotional energy, and personal resources, placing at its center the mother’s uninterrupted availability, her capacity to attune to the child’s needs, and to consistently prioritize them over her own.

This model risks exerting strong normative pressure on mothers, who may feel compelled to demonstrate their worth through caregiving practices perceived as “optimal,” such as prolonged breastfeeding, physical proximity, and immediate responsiveness. As a result, motherhood is represented as a totalizing experience, often difficult to reconcile with other domains of life and potentially a source of tension, ambivalence, and feelings of inadequacy when cultural expectations conflict with the material and relational conditions of everyday life.

Within this evolving literature, ECEC services remain a relatively underexplored context. Although several studies have documented the role of healthcare systems and workplace policies in supporting breastfeeding [7,23], far fewer investigations have examined how ECEC settings function as potential facilitators or constraints to breastfeeding continuation [11,24]. Yet, ECEC services represent a central caregiving environment in infants’ daily lives and a key site where caregiving practices, parental beliefs, and institutional norms intersect.

Emerging qualitative evidence from childcare settings suggests that on-site breastfeeding in ECEC contexts is often characterized by tensions between maternal expectations, educators’ practices, and organizational routines [24]. While educators frequently report positive attitudes toward breastfeeding, limited training, unclear policies, and rigid organizational structures may hinder the translation of supportive intentions into everyday practices [24,25]. At the same time, policy-oriented studies indicate that on-site breastfeeding-supportive environments in ECEC settings require formal organizational commitment, professional training, and a relational climate that recognizes the needs of both mothers and children [23,24].

In this light, breastfeeding beyond the home can be understood as a practice negotiated at the intersection of maternal beliefs, relational dynamics, and institutional contexts. ECEC services thus have the potential to function not only as logistical settings for childcare but also as relational and cultural environments that may either reinforce normative pressures or support diverse caregiving practices in non-prescriptive ways.

The present study addresses these gaps by examining on-site breastfeeding within ECEC settings as a relational and culturally embedded caregiving practice. Within educational research, family engagement and partnerships between parents and educators are recognized as core components of high-quality early childhood education [26,27]. Strong alliances between families and services promote children’s emotional security, support developmental continuity between home and educational settings, and foster shared educational projects [28,29]. From this perspective, breastfeeding can be understood not only as a nutritional practice but also as a relational and regulatory experience, supporting the child’s emotional well-being and facilitating separation processes during early childcare transitions.

These dimensions suggest the need to examine breastfeeding within ECEC services through an integrated lens that considers organizational practices, educator attitudes, parental beliefs, and broader cultural frameworks.

### Aims of the Current Study

This study presents an investigation conducted within the framework of a pilot parental engagement initiative aimed at enabling mothers of children attending ECEC services to continue breastfeeding, rather than discontinuing it, within the educational setting. The pilot study involved 17 ECEC services located in a large city in the Piedmont region of Northern Italy.

While existing literature has extensively examined the role of healthcare services in supporting breastfeeding, far fewer studies have systematically explored the role of ECEC services as potential facilitators or barriers to the continuation of breastfeeding. In this respect, the present study seeks to address this gap by examining ECEC services as educational and relational contexts in which caregiving practices and maternal feeding choices are actively negotiated and supported. The study aimed to pursue three main objectives:

- To analyze maternal beliefs regarding the importance of breastfeeding and to examine mothers’ personal breastfeeding experiences, with particular attention to feeding modality, duration, and the social support received from the partner. This analysis also considered potential differences according to maternal age, nationality, and educational level, as well as the age and gender of the child;
- To situate maternal attitudes toward breastfeeding within the theoretical framework of parental ethnotheories, examining their relationship with other early caregiving practices, particularly co-sleeping, in order to identify coherence or tensions between different cultural orientations to caregiving;
- To investigate the personal and contextual factors associated with the decision to continue breastfeeding within early childhood education and care settings, through a systematic comparison of caregiving beliefs and practices between mothers who chose to maintain breastfeeding in the educational service and those who, conversely, opted to discontinue it.

## 2. Materials and Methods

### 2.1. Procedures

Following approval from the University Ethics Committee, local nurseries were contacted and invited to participate in the study; participating nurseries subsequently distributed the self-report questionnaire to families. The study was conducted in the Piedmont region of northwestern Italy as part of a broader parental engagement initiative aimed at supporting the continuation of maternal breastfeeding within ECEC services. In the Piedmont region, awareness-raising initiatives aimed at supporting breastfeeding within ECEC services—both through on-site breastfeeding and through the provision of expressed breast milk administered by educational staff—were first introduced in 2018 and subsequently resumed following the COVID-19 pandemic; this regional trajectory aligns with the current National Prevention Plan of the Italian Ministry of Health and is consistent with recent World Health Organization guidelines, which emphasize the importance of community-based support for breastfeeding as a key practice in promoting child health and well-being.

To be eligible for participation, respondents were required to be mothers of children attending a nursery involved in the project. Informed consent was obtained via the introductory page of the questionnaire, which outlined the aims of the study and confirmed compliance with relevant ethical standards and data protection regulations. To ensure participants’ confidentiality, all responses were collected and analyzed anonymously. Data were collected between 2024 and 2025.

### 2.2. Measurements

Mothers completed:

- A socio-demographic questionnaire (age, nationality, educational level, marital status, number of children, age and gender of the child enrolled in the childcare center).
- An ad hoc closed-ended questionnaire designed to investigate mother’s beliefs regarding the importance of breastfeeding, co-sleeping, and the possibility of continuing breastfeeding within the child’s nursery. The questionnaire showed good face validity, as the items were grounded in previous literature and reviewed by experts in the field.
- Parenting Ethnotheory Scale [19] including 10 items measuring two dimensions of early childhood parenting: caregiving practices oriented toward promoting independence and caregiving practices oriented toward promoting interdependence. Mothers were asked to indicate their level of agreement with each item using a 5-point Likert scale (1 = strongly disagree; 5 = strongly agree). The Kaiser–Meyer–Olkin (KMO) measure of sampling adequacy was 0.726, and Bartlett’s Test of Sphericity was significant (χ^2^ = 629.90, df = 45, *p* < 0.001), indicating suitability for factor analysis. The factorial analysis of the scale confirmed the presence of the two factors (see Table 1): Factor 1 Independence (22.61% variance explained); Factor 2 Interdependence (21.21% variance explained). The internal consistency (Cronbach’s alpha) for the two subscales was α = 0.68, and α = 0.75, respectively. Although Cronbach’s alpha values below 0.70 are often considered suboptimal, methodological literature suggests that lower reliability coefficients can be acceptable in exploratory studies and in the assessment of complex, culturally embedded constructs. In particular, values around 0.60–0.70 have been deemed adequate in early-stage research and in scales capturing multidimensional orientations rather than homogeneous traits [30,31,32]. Moreover, Cronbach’s alpha is sensitive to the number of items included in a scale, and lower values are not uncommon in brief measures [33].

The Independence dimension included items which emphasize early self-regulation, autonomy, and separation, such as allowing brief periods of crying without immediate intervention, and promoting solitary sleep. These items reflect a caregiving orientation that prioritizes early independence and autonomous coping. In contrast, the Interdependence dimension comprised items which emphasize physical proximity, immediate responsiveness, and relational regulation. These items focus on practices such as promptly picking up a fussy infant, maintaining close bodily proximity to respond to the child’s signals, immediate nursing in response to crying, and caregiver-guided bodily practices (e.g., infant massage). Together, these items reflect a caregiving orientation grounded in interdependence, relational closeness, and shared emotional regulation. It is worth noting that the item “Gymnastics make a baby strong” loaded on the Interdependence dimension, a finding that may appear counterintuitive if physical exercise is interpreted as fostering autonomy. However, within the parental ethnotheory framework proposed by Keller [19], early motor stimulation is conceptualized as a caregiver-guided, body-based practice, embedded in close physical contact and relational regulation. In interdependent caregiving models, such practices aim to strengthen the child’s body for social integration rather than to promote independent exploration, thereby aligning more closely with interdependence-oriented socialization goals. In the Italian context, early motor stimulation practices such as infant massage are typically caregiver-guided and embedded in close bodily interaction. Rather than fostering early autonomy, these practices often reflect a relational orientation toward supporting the child’s development through physical proximity and adult-mediated regulation, consistent with interdependence-oriented ethnotheories.

Educators completed:

- A socio-demographic questionnaire (age, nationality, educational level, years of work experience).
- An ad hoc closed-ended questionnaire aimed at assessing their evaluation of the pilot experience that allowed mothers to breastfeed their children within the educational setting. The questionnaire showed good face validity, as the items were grounded in previous literature and reviewed by experts in the field.

### 2.3. Data Analysis

Data were analyzed using IBM SPSS Statistics (Version 29). Missing data were handled using available-case analysis, such that cases with missing values were excluded only from the specific analyses for which the relevant variables were missing. Consequently, sample sizes vary across analyses depending on the variables considered. Descriptive statistics were computed to examine the distribution of all study variables.

To investigate associations between maternal beliefs and practices related to breastfeeding and parenting ethnotheories, a combination of analyses of variance (Oneway ANOVA), Pearson’s chi-square tests and binary logistic regression was employed.

### 2.4. Participants

The study sample consisted of 490 mothers, aged between 20 and 54 years (M = 35.84; SD = 5.09). The majority of participants were of Italian nationality (68.5%). With regard to educational level, 19.9% of mothers had a low level of education (below upper secondary school), 31.1% had a medium level of education (secondary school diploma), and 49% had a high level of education (university degree or postgraduate qualification).

In terms of family status, 93% of participants were married or cohabiting with a partner, while 4.5% were not in a stable partnership; 48.1% of mothers were experiencing their first pregnancy, while 51.9% had experienced more than one pregnancy.

The children currently attending ECEC services were male in 52.7% of cases and female in 47.3%. Children ranged in age from 6 to 30 months (M = 25.21; SD = 6.50). In 77% of cases, children attended ECEC services on a full-time basis.

A total of 118 educators also took part in the study; all were female and of Italian nationality, with a mean age of 46.21 years, a predominantly medium-to-high level of education, and extensive professional experience (M = 17.08 years; SD = 11.20).

The sociodemographic characteristics of the sample are presented in Table 2.

## 3. Results

### 3.1. Breastfeeding and Early Childhood Care Practices: Maternal Beliefs, Parental Ethnotheories, and Social Support

Mothers’ beliefs regarding breastfeeding were found to be strongly oriented toward recognizing its benefits for the child. When asked whether they considered breastfeeding to be preferable for the child’s physical and emotional development, whether there were no differences between breastfeeding and formula feeding, or whether formula feeding was preferable, 85.5% of participants stated—regardless of their personal experience—that breastfeeding is preferable for the child’s psychophysical development. Within this group, 13.1% considered it desirable to continue breastfeeding up to 6 months, 28% up to 1 year, and 41% beyond 12 months of age, while 18% did not provide a response to the question concerning the age up to which they considered breastfeeding to be important.

In addition to maternal beliefs about breastfeeding, we also examined mothers’ personal experiences by asking whether they had actually breastfed and for how long. Seventy-six point five percent of mothers reported having exclusively breastfed, 13.7% reported mixed feeding with supplementation of infant formula, and 9.8% reported that they were unable to breastfeed or had to discontinue breastfeeding early due to maternal health issues or infant-related difficulties. Among mothers who breastfed, 74.2% reported being able to continue breastfeeding in accordance with their own preferences, discontinuing when the child began to show interest in other foods. On average, breastfeeding continued until nearly the child’s first year of life (Min = 1 month; Max = 42 months; M = 11.55 months; SD = 7.50). The standard deviation indicates a moderate degree of variability in the timing of breastfeeding cessation across the sample.

Partner support was assessed by asking mothers to report the attitude of the child’s father toward their decision to breastfeed, selecting one response among the following options: actively supportive, favorable but insufficiently supportive, indifferent, or openly opposed. The data indicate a predominance of supportive and favorable attitudes toward breastfeeding. Seventy-three point three percent (73.3%) of mothers reported that breastfeeding was the outcome of a shared decision with their partner, from whom they perceived active support. A smaller proportion (7.6%) reported that, despite the partner’s favorable attitude toward breastfeeding, no concrete support was provided. Four point one percent (4.1%) of mothers described an attitude of substantial disinterest on the part of the partner, while only 0.6% reported an attitude of open opposition to breastfeeding.

Overall, these findings suggest that the partner represents, in most cases, a significant relational resource in supporting breastfeeding practices. At the same time, the data highlight that the quality of partner support may vary considerably, ranging from active and engaged involvement to more passive or marginal forms of support.

As breastfeeding choices are often associated with co-sleeping practices, mothers were asked to report their views on co-sleeping and infant sleep arrangements. Co-sleeping practices were assessed by asking mothers where they believed it was best for their child to sleep, with response options including sleeping in the same room as the mother (either in a crib/cot next to the parental bed or in the parental bed) or sleeping in a separate room. The motivations underlying co-sleeping choices were also examined. Seventy-eight point three percent of mothers reported that a child of this age should sleep in the same room as the mother, either in a crib placed next to the parental bed (73.3%) or in the parental bed (26.7%). The main reasons reported in support of co-sleeping included: the provision of comfort and emotional closeness to the child (55.6% of responses); the facilitation of breastfeeding (54% of responses); the strengthening of the mother–child emotional bond (48.4% of responses); the perception of co-sleeping as a safer option for the child (34.4% of responses). In contrast, mothers who considered it more appropriate for the child to sleep in a separate room from the parent primarily justified this choice by: concerns about being disturbed by the child’s presence during sleep (37.9% of responses); the belief that co-sleeping might make it more difficult at a later stage to accustom the child to sleeping independently in their own room (64.8% of responses).

As early caregiving practices related to infant feeding and sleep reflect specific parental ethnotheories, we also examined the extent to which mothers in the sample endorsed parenting practices oriented toward the promotion of early child independence or, conversely, toward the promotion of relational interdependence (see Table 3).

Overall, comparisons between scores on the *Independence* and *Interdependence* scales indicate that mothers expressed a higher level of agreement with caregiving practices oriented toward the promotion of interdependence, physical proximity to the child, and primary intersubjectivity. These practices emphasize the importance of responding promptly to infants’ signals of distress through physical contact and breastfeeding, understood as key mechanisms of emotional regulation and relational engagement.

Notably, however, among practices more strongly oriented toward independence, mothers reported a significant level of agreement with the item *You cannot start early enough to direct the infant’s attention towards objects and toys* referring to the caregiver’s role as a “presenter of objects”. This finding highlights the acknowledged relevance of secondary intersubjectivity, suggesting a caregiving representation that, while prioritizing relational closeness, also incorporates elements of mediation between the child and the external world and supports the child’s gradual orientation toward autonomy.

### 3.2. Breastfeeding Beyond the Home: Maternal Perspectives on Breastfeeding in the ECEC Services

With regard to breastfeeding within ECEC services, we examined whether mothers had been informed about the possibility of continuing breastfeeding at the nursery, either through on-site breastfeeding or by providing expressed breast milk, and by whom this information had been provided. Sixty-four point five percent (64.5%) of mothers in the sample reported having been informed about the possibility of continuing breastfeeding within ECEC services, whereas 35.5% reported not having received any information on this option. Educators emerged as the primary source of information (63.8% of cases), followed by other professional figures within the service (34.2% of cases), such as pedagogical coordinators or administrative staff; in some instances, information was also conveyed by other parents or by local healthcare service staff.

Despite having received information, 66.1% of mothers chose not to continue breastfeeding at the childcare center, while 33.9% decided to maintain on-site breastfeeding within the service. Among the latter, in response to the question regarding at which time of day they preferred to breastfeed their child at the nursery, breastfeeding most commonly occurred in the morning at the time of separation (71.6%) and in the afternoon during reunification with the child (72.6%). Only 9.6% of mothers who chose to continue breastfeeding within the childcare setting reported returning to the nursery multiple times during the day to breastfeed their child.

We also examined the reasons why mothers chose to continue breastfeeding at the nursery. Mothers were asked whether they considered the experience of breastfeeding at the nursery to be more beneficial for the child, for themselves, or for both, and to specify the reasons underlying this evaluation; multiple responses were allowed. Mothers who continued breastfeeding within the ECEC services tended to interpret this choice as particularly beneficial for the child (74.2% of responses), especially with regard to managing the separation from the mother. In particular, they emphasized how breastfeeding helped to reduce the sense of separation (69% of responses) and to promote greater emotional security for the child within the educational context (71.1% of responses).

Mothers who reported that continuing breastfeeding was primarily beneficial for themselves (50.8% of responses) highlighted instead its function as a form of emotional support for the mother (46.6% of responses), noting that this practice made the management of separation and the child’s transition into the childcare setting less demanding (72.4% of responses).

Taken together, these findings suggest that continuing breastfeeding within the educational service fulfills not only a nutritional function, but also a relational and regulatory function, benefiting both the child and the mother.

Finally, mothers were asked to indicate which factors had facilitated their decision to continue breastfeeding at the nursery, selecting among the organizational climate of the childcare center (e.g., the presence of other breastfeeding women, comfortable environments, information provided by educational staff), support from the educational team, and their own personal beliefs (e.g., the desire not to discontinue breastfeeding). According to mothers’ reports, the decision to continue breastfeeding within ECEC services was primarily influenced by personal beliefs (56.4% of responses), followed by the perception of a welcoming and supportive organizational climate within the childcare setting (52.6% of responses) and by active and explicit support from educators (39.1% of responses). These findings indicate that the choice to maintain breastfeeding at the nursery is shaped by the interplay between individual value orientations and the relational characteristics of the educational context.

Subsequent analyses aimed to examine the variables associated with the decision to continue breastfeeding on-site at the nursery, by dichotomizing the outcome variable into Yes (regardless of the time of day) and No. Mothers who chose to continue on-site breastfeeding at the childcare center actually showed higher mean scores on parental ethnotheory scales emphasizing physical and relational proximity to the child, outlining a coherent set of beliefs regarding early caregiving practices. Indeed, ANOVA one-way analysis revealed significant differences in the mean scores of the two Parenting Ethnotheory Scale dimensions, with an effect size ranging from small to moderate (see Table 4).

Maternal age did not emerge as a significant variable, whereas the child’s age at nursery entry played a relevant role: the younger the child, the more likely breastfeeding was to be continued within the educational setting (Pearson chi-square = 37.69; *p* ≤ 0.001), with no differences between males and females.

Consistent with this orientation, mothers who continued on-site breastfeeding at the nursery reported higher rates of co-sleeping compared to mothers who did not continue breastfeeding in the childcare setting (84.7% vs. 72.9%, respectively; Pearson chi square = 7.10; *p* ≤ 0.001). Finally, no significant differences emerged between Italian and non-Italian mothers in the decision to continue breastfeeding at the nursery, suggesting that this choice is less associated with nationality and more closely related to caregiving beliefs and practices.

Finally, in order to further examine the relative contribution of each variable investigated, a binary logistic regression (see Table 5) was conducted to examine predictors of breastfeeding continuation at the nursery. In the model, the decision to continue breastfeeding on-site at the nursery (yes/no) was used as the outcome variable, and the following variables were included as covariates: Maternal nationality (dichotomized: Italian; No Italian), child age (dichotomized: <1 year; >1 years), educational level (three-category categorical variable: Low; Medium; High), and partner support (dichotomized: absent; present). The overall model was statistically significant (Chi square = 28.32; *p* < 0.001; Nagelkerke R^2^ = 0.10) and correctly classified 66.8% of cases.

Higher interdependence-oriented ethnotheory scores significantly increased the likelihood of continuing breastfeeding (OR = 1.46, 95% CI [1.10, 1.95], *p* = 0.010), whereas higher independence-oriented scores significantly decreased the likelihood of breastfeeding continuation (OR = 0.61, 95% CI [0.47, 0.80], *p* < 0.001). Maternal nationality, educational level, and partner support were not significant predictors in the model, suggesting that cultural caregiving orientations played a more central role than socio-demographic variables in shaping breastfeeding continuation within ECEC settings.

### 3.3. Breastfeeding Beyond the Home: Educators’ Perspectives on Breastfeeding in the ECEC Services

Overall, professionals’ evaluations of the breastfeeding experience within ECEC services were largely positive. Seventy-three point nine percent of the educational staff reported having provided mothers with information about the possibility of continuing breastfeeding at the nursery, indicating that this most often occurred during the family’s first visit to the childcare center or during the meeting with parents prior to the child’s enrollment. In response to the question of whether they considered on-site breastfeeding to be more functional at the time of morning separation, during afternoon reunification, or at any time during the day at the nursery, educators reported that breastfeeding was particularly functional in the morning at the time of arrival (80.5%) and during reunification (75.2%), while recognizing its more limited functionality at other times of the day, although still viewing it as an option that should remain available to mothers.

In addition, educators were also asked whether they considered the experience of breastfeeding at the nursery to be more beneficial for the child, for the mother, or for both, and to specify the reasons underlying this evaluation; multiple responses were allowed. According to professionals, allowing breastfeeding at the nursery is a practice that is beneficial for both the mother and the child (75.2% of responses). Specifically, they reported that breastfeeding helps mothers feel more calm and reassured (52.6% of responses) and facilitates the separation process from the child (89.7% of responses). At the same time, professionals acknowledged benefits for the child, who may feel more secure (76.8% of responses) and experience less distress during separation (61% of responses).

From an educational and relational perspective professionals evaluated breastfeeding at the nursery by expressing their level of agreement with nine items on a five-point Likert scale (see Table 6).

Educators reported that breastfeeding at the nursery is a meaningful way of responding to the needs of the mother–child dyad and as a practice that supports the strengthening of the affective bond. Furthermore, they recognized that this experience contributes to promoting parental involvement in the life of the service and to developing a deeper understanding of both the mother and the child. Negative evaluations of the experience received very low scores. Professionals reported only minimal agreement with the idea that breastfeeding at the nursery represents an additional burden for staff, a source of disturbance for other children, or an obstacle to the regular flow of the educational day. Overall, these findings suggest that, from professionals’ perspectives, breastfeeding can be effectively integrated into everyday nursery routines without compromising educational organization, provided that it is embedded within a welcoming and shared relational climate. This finding should nevertheless be interpreted in relation to the fact that the study was conducted in ECEC settings that had taken part in the pilot initiative promoting breastfeeding at the nursery.

## 4. Discussion

The present study provides new insights into the cultural and relational dynamics of breastfeeding support in ECEC settings. In line with global evidence that returning to work or enrolling in childcare often precipitates early weaning [7,34,35], our findings highlight that a substantial minority of mothers (about one-third) continued breastfeeding at the nursery when given an enabling environment. Data from the overall sample indicate that, on average, breastfeeding was discontinued around the child’s first year of life. According to mothers’ reports, this was often due to the child’s emerging interest in new tastes and textures, but also to concerns that breast milk might no longer be sufficient to support adequate growth, to the fatigue associated with breastfeeding in combination with work schedules, and, in some cases, following the pediatrician’s recommendation.

However, nearly 85% of mothers in our sample believed that breastfeeding is optimal for their child’s development. This strong endorsement of breastfeeding’s importance is consistent with public health literature underscoring its dose-dependent benefits for maternal and child health [36,37]. Our study extends this knowledge by demonstrating that ECEC institutions can serve as facilitators—rather than mere obstacles—to continued breastfeeding, provided that supportive policies and attitudes are in place [24]. This addresses a notable gap in the literature: while much research has focused on healthcare and workplace supports for breastfeeding, far less attention has been given to the role of ECEC services. By examining breastfeeding in the nursery through both maternal and educator perspectives, we contribute a nuanced understanding of how early education settings can align with family feeding goals, thereby promoting continuity of care for infants and toddlers. Consistent with our theoretical framework, mothers’ feeding decisions and practices were deeply intertwined with broader parental ethnotheories and cultural models of childrearing. Parental ethnotheories are the culturally shaped beliefs that define parents’ roles and goals for their children [19,22], and in our sample these beliefs clearly favored a proximal, child-centered orientation. Most mothers emphasized responsiveness and physical closeness in caregiving—for example, 78% endorsed co-sleeping (room-sharing or bed-sharing) with their infant, frequently citing the desire to provide comfort, emotional security, and to facilitate night-time breastfeeding. Such preferences align closely with the parenting practices observed in “proximal care” cultures, where infants remain in close contact and feed on demand, in contrast to “distal” caregiving that emphasizes infant independence and scheduled routines. Indeed, research has shown that co-sleeping and other proximal practices tend to support longer breastfeeding duration, whereas separating infants at night is associated with earlier weaning. Our findings mirror this pattern: mothers in our study overwhelmingly valued prompt responsiveness (e.g., immediately tending to a crying baby) and maintained near-constant physical proximity through practices like breastfeeding and co-sleeping. This pattern can be interpreted within the broader theoretical framework of intensive mothering [38], which conceptualizes contemporary motherhood as characterized by high levels of time, emotional investment, and responsiveness to children’s needs. Within this perspective, practices such as extended breastfeeding, co-sleeping, and babywearing are discussed in the literature as potential expressions of caregiving orientations that prioritize proximity and relational attunement. While the present findings do not allow for causal or identity-based inferences, they suggest that some maternal practices observed in the sample may be meaningfully situated within cultural models that emphasize intensive and relational forms of caregiving. Notably, however, we also detected a subtle blend of orientations: for instance, mothers showed some agreement with encouraging early autonomy (e.g., introducing toys to stimulate infants), indicating that Italian parenting norms may integrate both traditional relational values and certain autonomy-promoting goals. This nuanced combination of proximal and distal elements is in line with prior cross-cultural research showing that parenting beliefs in societies undergoing cultural transition often reflect both interdependence and independence goals [19,20]. Overall, our results confirm that maternal attitudes toward breastfeeding and infant care are grounded in culturally sanctioned ethnotheories—in this case, a predominant belief in the primacy of maternal–infant closeness and responsiveness—which provide a context for interpreting the specific feeding choices mothers make.

Within this cultural context, a key contribution of our study is the identification of clear differences between mothers who continued breastfeeding at the nursery and those who did not, in terms of their beliefs, practices, and certain demographics. Mothers who chose to maintain breastfeeding in the ECEC services displayed a distinctly coherent set of caregiving beliefs aligned with a proximal care orientation. They scored significantly higher on the parental ethnotheory scale for relational interdependence (emphasizing immediate response, holding, and comforting) and lower on the independence dimension, compared to mothers who weaned upon entering childcare. In practical terms, virtually all breastfeeding-continuing mothers were also co-sleepers: 84.7% reported parent–infant bedsharing or room-sharing, a rate notably greater than the 72.9% observed among non-breastfeeding mothers. This association between co-sleeping and sustained breastfeeding corroborates the idea that these behaviors stem from a consistent caregiving ethos; mothers most committed to maintaining a physical and emotional bond—day and night—were the ones most likely to extend nursing into the nursery context. These findings echo prior studies linking attachment parenting practices (such as long-term breastfeeding on demand, bed-sharing, and infant carrying) with an underlying intensive mothering ideology. By contrast, mothers who discontinued breastfeeding upon childcare enrollment tended to exhibit a somewhat more distal orientation on average—for example, they were less likely to share sleep and somewhat more accepting of infant autonomy—though it is important to note that even this group largely acknowledged breastfeeding’s benefits and practiced it for many months before weaning.

Within this relational framework, caregiving beliefs do not operate at the individual level alone but are negotiated within the parental dyad, highlighting the role of the partner as a key cultural mediator in breastfeeding decisions.

Findings related to partners’ attitudes toward breastfeeding and to maternal beliefs about its value can be further interpreted through the lens of parental ethnotheories. From this perspective, the partner does not merely function as a source of emotional or practical support, but plays a meaningful role as a cultural mediator in the construction, legitimization, and negotiation of parental choices.

The high proportion of mothers who described breastfeeding as a shared decision supported by their partner suggests the presence of relatively coherent parental ethnotheories within the couple, in which breastfeeding is recognized as an appropriate and desirable caregiving practice for the child’s well-being. In these cases, partner support appears to reinforce an understanding of breastfeeding as a relational practice, helping to reduce maternal isolation and to legitimize the emotional and organizational investment required to sustain breastfeeding over time.

Conversely, situations in which partners expressed general approval of breastfeeding but failed to provide concrete support, or showed disinterest, may reflect partially misaligned ethnotheories, in which breastfeeding is acknowledged as an abstract value but not fully integrated into everyday caregiving practices or shared representations of parental roles. In such contexts, mothers may experience breastfeeding as a more solitary endeavor, carrying a greater emotional and organizational burden, with potential implications for their subjective experience and for the continuity of the practice.

The widespread maternal endorsement of breastfeeding as superior for children’s psychophysical development, together with the desire to continue breastfeeding beyond the first year of life, further points to ethnotheories strongly oriented toward maternal responsiveness and relational continuity. However, the discrepancy observed between these beliefs and mothers’ actual decisions to continue breastfeeding within the childcare setting highlights that ethnotheories do not operate in isolation. Rather, they interact with other systems of norms and expectations, including those embedded in institutional and educational contexts. The decision to continue or discontinue breastfeeding at the nursery can thus be understood as the outcome of a complex negotiation between personal convictions, relational support within the couple, and perceived compatibility between family caregiving practices and the educational context [23,39,40].

Interestingly, maternal nationality did not significantly differentiate those who breastfed at the nursery from those who did not. The lack of a nationality effect suggests that within our sample the decision to continue breastfeeding was less about country-of-origin culture and more about individual orientations and the enabling conditions. In other words, immigrant and Italian-born mothers were equally inclined to pursue on-site nursing when they held strong proximal care beliefs and had support to do so. This finding contrasts with some global research showing cultural differences in feeding practices, and implies that a supportive childcare context can bridge diverse cultural backgrounds, allowing personal mothering values to be the driving force. It is also possible that the mothers self-selecting into this pilot project (and the centers that participated) shared a certain openness to intensive parenting values, regardless of heritage. Future research employing focus group methodology could further explore how processes of acculturation and cultural background interact with such initiatives.

Crucially, educators’ perspectives and characteristics of the educational context appear to play a meaningful role in shaping conditions that may either facilitate or constrain the continuity of breastfeeding among mothers. Our results paint an overwhelmingly positive picture of educator attitudes in the pilot project: childcare professionals not only informed mothers of the option to breastfeed on-site, but also actively welcomed and accommodated this practice. Educators recognized multiple relational benefits of allowing breastfeeding at the nursery—for the child, the mother, and the educator–family relationship. They observed that breastfeeding at drop-off and pick-up times helped children feel more secure and eased the stress of separation. From the mothers’ side, staff noted that the opportunity to nurse left mothers feeling calmer and more reassured during the day. In essence, professionals viewed on-site breastfeeding as a meaningful way to respond to the needs of the mother–infant dyad and to smooth the attachment disruption inherent in daily separations. This is an important contribution because it challenges any assumption that breastfeeding might “interfere” with the institutional routine; on the contrary, our participating educators largely felt that it strengthened the affective bond and even enhanced parental involvement in the center’s life. Quantitatively, they rated potential negatives (e.g., breastfeeding causing extra work, disturbance to other children, or schedule disruptions) as very low concerns. These findings highlight the value of the educational context as a potential active facilitator in breastfeeding continuance. The nursery, when characterized by a welcoming climate and shared relational understanding, can integrate maternal practices like breastfeeding into its daily flow without compromising educational activities. Moreover, the fact that mothers tend to prefer breastfeeding in the morning at the time of separation and in the afternoon during reunification makes breastfeeding at the nursery more compatible with the organizational constraints of the educational context. This aligns with the concept of continuity of care and family-centered practice in early education: respecting and incorporating home routines and practices to create consistency for the child. It is noteworthy that our data reflect a best-case scenario of sorts—a pilot initiative where centers were presumably motivated and perhaps guided to support breastfeeding. In less proactive environments, attitudes might differ. For instance, a recent US-based study found that early childhood educators felt neutral or only modestly supportive of breastfeeding in childcare, often citing constraints such as strict infant feeding regulations, lack of training, or time pressures as barriers [13]. Some providers also underestimated the demand or importance of such support, perceiving it as a low priority for families. By contrast, our study suggests that when educators are adequately informed and philosophically on board, they do not view maternal on-site breastfeeding as a burden—an important proof of concept. The educators’ largely positive stance likely reflects both their professional ethos of meeting children’s emotional needs and the specific training or orientation provided by the project. It may also indicate that Italian ECEC culture, especially in this region, embraces a relational pedagogy that values close partnership with parents (as evidenced by Italy’s strong tradition of family–educator collaboration in infant-toddler centers). In this light, breastfeeding at the nursery can be seen as an extension of responsive caregiving: just as educators are expected to respond to each child’s needs sensitively, here they extended that responsiveness to facilitating the mother’s presence and milk as needed. This finding further highlights the potential importance of contextual factors, suggesting that mothers who might otherwise discontinue breastfeeding in less supportive circumstances may be more inclined to continue when the educational environment is perceived as encouraging and accommodating. It points to the possibility that breastfeeding in childcare settings may be understood not only as an individual maternal commitment, but also as a relational practice that can be sustained through the involvement of a broader caregiving network, including professional caregivers. At the same time, the regression analysis suggests that maternal beliefs about caregiving practices are more strongly associated with the likelihood of continuing breastfeeding at the nursery, indicating that cultural orientations may play a central role alongside, rather than being overridden by, contextual support.

Furthermore, the opportunity to continue breastfeeding with the support of professional caregivers suggests that practices often associated with intensive mothering—such as extended and out-of-home breastfeeding—may not necessarily constitute an “all-or-nothing” commitment. Rather, they can be understood as part of a shared caregiving project involving multiple supportive actors, which may help to attenuate some of the potential risks associated with intensive mothering, including maternal exhaustion and social isolation [41,42].

### Limitations and Future Directions

Despite its contributions, this study has several limitations that should be acknowledged:

Sample Characteristics and Representativeness: The sample was drawn from 17 early childcare centers in a single metropolitan area of the Piedmont region in Northern Italy. Participants were predominantly Italian mothers with relatively high education levels, and the vast majority were in stable partnerships. This context-specific and self-selected sample may not be fully representative of the broader population of mothers. Caution is warranted in generalizing the findings to families in different regions, to single mothers or less-educated populations, or to cultural contexts outside of Italy.

Contextual and Cultural Specificity: The research took place within a pilot program in Italy that actively encouraged breastfeeding in ECEC settings. This supportive institutional climate likely influenced the attitudes and high acceptance observed. Consequently, the results may not readily transfer to contexts with different childcare policies or cultural norms. In countries or settings where on-site breastfeeding is not supported or culturally accepted, mothers’ and educators’ perspectives might diverge significantly from those reported here.

Observational Cross-Sectional Design: This study was observational and cross-sectional in nature, which limits the ability to infer causality or developmental trends. Mothers self-determined whether to continue breastfeeding at the nursery or not, so any differences found between those who did and did not continue could partly reflect pre-existing characteristics rather than effects of the breastfeeding practice itself. Furthermore, no longitudinal data were collected to track changes over time. Without follow-up, we cannot determine long-term outcomes or whether initial attitudes and behaviors (e.g., maternal ethnotheories or child adjustment) changed as a result of the breastfeeding-in-childcare experience. Future research using longitudinal designs or control groups would help clarify the direction of these associations.

Self-Report Biases: All data were gathered through self-report questionnaires, relying on mothers’ and educators’ own perceptions and recall. This methodology entails inherent limitations such as social desirability bias and recall bias. For example, mothers may have overestimated the positive aspects of breastfeeding or the level of partner support, while educators may have downplayed potential difficulties due to the sensitive and value-laden nature of breastfeeding practices, as well as to self-selection bias associated with voluntary participation by highly motivated professionals. The lack of objective measures or observations (e.g., direct assessments of child well-being or independent verification of breastfeeding frequency) means the findings are based on subjective reports, which should be interpreted with appropriate caution.

Theoretical and Measurement Scope: The study’s theoretical focus was mainly on parental ethnotheories (independence vs. interdependence orientations) to contextualize caregiving practices. While this framework is useful, it may not capture all relevant factors influencing the decision to continue breastfeeding. Other psychosocial factors—such as attachment security, maternal mental health, workplace constraints, or detailed socio-economic variables—were not examined in depth.

Each of these limitations suggests directions for future research. Acknowledging these limitations does not detract from the value of the study; instead, it provides a balanced view and underscores the context within which the results should be understood.

For research, this study opens several avenues. Given that it was a pilot initiative in one cultural context, cross-cultural research is needed to see how these dynamics unfold elsewhere—for instance, in countries with different maternity leave lengths, breastfeeding norms, or childcare systems. Comparative studies could examine whether the strong association we found between co-sleeping, ethnotheories, and nursing at childcare holds in other societies, or whether different factors come into play. Longitudinal research could also explore the long-term effects of continuing breastfeeding in childcare on child adjustment, mother–child attachment, and maternal well-being. Furthermore, future studies could investigate the role of paternal or partner support in this equation, comparing maternal and paternal beliefs and attitudes: We documented that most partners were supportive of breastfeeding in general, but it remains unexplored how partners view on-site breastfeeding or how their involvement might influence outcomes. Understanding these hurdles can inform more tailored interventions. Finally, building on the educator perspective, research should probe how we can support childcare staff who may initially feel unprepared for this role.

In conclusion, the present study’s relational and cultural analysis demonstrates that continuing breastfeeding in ECEC is not only feasible but mutually beneficial—reinforcing child–mother bonds, empowering mothers, and enriching the educational context.

## 5. Conclusions: Implications for Practice and Policy

The findings of this study highlight the significant role that ECEC services can play in supporting the continuation of breastfeeding, thereby contributing to child well-being and to the overall quality of the educational experience. From this perspective, several relevant implications emerge for educational practice, service organization, and public policy.

First, ECEC services can be recognized as key contexts for the promotion of breastfeeding, not only in logistical terms but, above all, in relational and cultural ones. The opportunity to breastfeed within childcare settings, when supported by a welcoming organizational climate and shared practices, promotes continuity of care and supports children’s processes of separation and adaptation. This underscores the importance of explicitly integrating breastfeeding support into the organizational guidelines and quality frameworks of early childhood services.

Second, the central role of educators clearly emerges. Initial and in-service training should include specific content on breastfeeding that goes beyond biological aspects and encompasses emotional, relational, and cultural dimensions. Educators who are informed, reflective, and collectively oriented toward supporting breastfeeding can help create a coherent educational environment, reducing ambiguity and discontinuity in communication with families.

A further implication concerns family engagement. Providing parents with early and clear information about the possibility of continuing breastfeeding within childcare services, fostering listening practices, and valuing parental competencies contribute to the development of stronger educational partnerships. From this perspective, breastfeeding can become a privileged space for dialogue between families and services, supporting the construction of a shared educational project that is respectful of cultural diversity and individual choices. In this regard, the role of educators is crucial. Initial and ongoing professional training should include relational and reflective competencies that enable practitioners to recognize the complexity of maternal experiences and to avoid implicitly blaming or idealizing approaches. Educators equipped with such competencies can better support families by promoting informed, flexible, and culturally sensitive choices. Through attentive and non-judgmental communicative practices, ECEC services can contribute to creating spaces in which mothers feel authorized to express fatigue, ambivalence, and the desire to negotiate between their own needs and those of the child.

From a policy standpoint, the findings point to the need for greater integration between educational and health systems. Breastfeeding support should not be delegated exclusively to healthcare services but recognized as a shared responsibility that also involves early childhood education services. Early childhood-oriented welfare policies could encourage breastfeeding-friendly practices within ECEC settings by promoting quality standards that include support for parenting and continuity of care. From a policy perspective, the findings highlight the importance of promoting a conception of breastfeeding support that does not function as a moral imperative, but rather as a right accompanied by enabling conditions. Integrated educational and healthcare policies should acknowledge that child well-being is closely interconnected with maternal well-being, and that effective support practices are those capable of balancing relational care, emotional sustainability, and family quality of life.

Finally, from a practical standpoint, the findings suggest that “breastfeeding-friendly” ECEC services may be supported through a set of minimum policy elements. These include the explicit recognition of breastfeeding as a legitimate practice within the service, the availability of designated or clearly authorized spaces for on-site breastfeeding and the handling of expressed breast milk, and the adoption of clear communication protocols that inform families about these possibilities prior to enrollment. In addition, basic staff training focused on the relational and non-normative dimensions of breastfeeding support may help ensure consistent and non-judgmental practices across educators.

Importantly, implementation strategies should be tailored to the child’s age. Given the strong association between younger age and breastfeeding continuation, supportive measures appear particularly relevant during the early months of nursery attendance and transitional phases, such as initial separation and daily reunification. As children grow older, breastfeeding support may require greater flexibility and individualized negotiation, rather than standardized routines.

Taken together, these elements highlight how ECEC services can move beyond symbolic endorsement toward concrete, developmentally sensitive practices that support breastfeeding continuity while respecting family diversity and maternal well-being.

## Figures and Tables

**Table 1:** Exploratory Factor Analysis: Parenting Ethnotheory Scale.

	Factor Loadings	
	1	2
Sleeping through the night should be trained as early as possible	**0** **.644**	−0.070
You cannot start early enough to direct the infant’s attention towards objects and toys	**0** **.619**	0.247
Babies should be left crying for a moment in order to see whether they console themselves	**0** **.619**	−0.185
It is not necessary to react immediately to a crying baby	**0** **.585**	−0.300
It is good for a baby to sleep alone	**0** **.540**	−0.362
If a baby is fussy, he/she should be immediately picked up	−0.085	**0** **.740**
A baby should be always in close proximity with his/her mother, so that she can react immediately to his/her signals	−0.053	**0** **.688**
When a baby cries, he/she should be nursed immediately	0.049	**0** **.678**
Gymnastics make a baby strong	0.427	**0** **.608**
It is important to rock a crying baby on the arms in order to console him/her	−0.253	**0** **.370**

**Table 2:** Sociodemographic characteristics of the sample: Mothers, Children and Educators.

	N	Valid %/M *(SD)*
**Mothers**		
*Age* Missing	487 3	35.84 *(5.09)*
*Nationality:* Italian Non-Italian Missing	332 153 4	68.5 31.5
*Level of education:* Low Medium High Missing	97 152 239 2	19.9 31.1 49.0
*Marital status:* Cohabiting/married Single Divorced Widowed Missing	450 22 11 1 6	93.0 4.5 2.3 0.2
*Pregnancy:* First pregnancy Second or subsequent pregnancy Missing	228 246 16	48.1 51.9
**Children**		
*Sex:* Male Female Missing	251 225 14	52.7 47.3
*Age at nursery entry:* <6 months 6–12 months 12–24 months >24 months Missing	19 195 226 37 13	4.0 40.9 47.4 7.8
*Nursery attendance:* Part-time Full-time Missing	112 375 3	23.0 77.0
*Age at assessment (months):* Missing	486 4	25.21 *(6.50)*
**Educators**		
*Age* Missing	116 2	46.21 *(11.78)*
*Sex:* Female Male Missing	118 0 0	100 0 0
*Years of work experience* Missing	110 8	17.08 *(11.20)*
*Level of education:* Low Medium High Missing	21 60 32 5	18.6 53.1 28.3

**Table 3:** Parenting Ethnotheory Scale: Mothers.

ITEM	N	MIN	MAX	M	SD
Sleeping through the night should be trained as early as possible	472	1	5	3.00	1.45
You cannot start early enough to direct the infant’s attention towards objects and toys	466	1	5	3.28	1.24
Babies should be left crying for a moment in order to see whether they console themselves	474	1	5	2.64	1.31
It is not necessary to react immediately to a crying baby	474	1	5	2.31	1.24
It is good for a baby to sleep alone	475	1	5	3.03	1.36
*Total scale Independence*	*481*	*1*	*5*	*2.85*	*0.85*
If a baby is fussy, he/she should be immediately picked up	471	1	5	2.75	1.30
A baby should be always in close proximity with his/her mother, so that she can react immediately to his/her signals	472	1	5	2.97	1.43
When a baby cries, he/she should be nursed immediately	473	1	5	2.01	1.13
Gymnastics make a baby strong	469	1	5	3.14	1.33
It is important to rock a crying baby on the arms in order to console him/her	471	1	5	2.75	1.30
*Total scale Interdependence*	*482*	*1*	*5*	*3.02*	*0.77*

**Table 4:** Breastfeeding at the childcare center and parental ethnotheories.

Breastfeeding at the Childcare Center	Independence Scale M *(SD)*	Interdependence Scale M *(SD)*
Yes	2.61 *(0.85)*	3.18 *(0.79)*
No	2.96 *(0.83)*	2.91 *(0.72)*
*Total* **F (*p.*)**	2.84 *(0.85)* **16.40 (<0.001)**	3.00 *(0.76)* **12.32 (<0.001)**
**η^2^**	**0.06**	**0.05**

**Table 5:** On-site breastfeeding at the nursey: Binary logistic regression.

Predictor	B	SE	Wald	df	*p*	OR (Exp(B))	95% CI for OR
Child age	−0.773	0.542	2.032	1	0.154	0.462	[0.159, 1.336]
Maternal nationality	0.400	0.264	2.291	1	0.130	1.491	[0.889, 2.502]
Educational level (overall)	—	—	3.912	2	0.141	—	—
Educational level (Level 1)	0.651	0.356	3.347	1	0.067	1.917	[0.955, 3.851]
Educational level (Level 2)	0.639	0.344	3.455	1	0.063	1.895	[0.966, 3.718]
Interdependence	0.380	0.148	6.617	1	**0** **.010**	**1.462**	**[1.095, 1.953]**
Independence	−0.487	0.134	13.272	1	**<** **0** **.001**	**0.614**	**[0.473, 0.798]**
Partner support	−0.218	0.308	0.501	1	0.479	0.804	[0.439, 1.471]
Constant	−0.248	0.884	0.079	1	0.779	0.780	—

Notes: Outcome variable: on-site breastfeeding continuation at the nursery (0 = No, 1 = Yes). OR = Odds Ratio; CI = Confidence Interval. Educational level was included as a three-level categorical variable. Significant predictors are highlighted in bold.

**Table 6:** Educators: evaluation of breastfeeding at the nursery.

Breastfeeding at the Nursery Is	M	SD
A valuable opportunity for mothers and children to maintain their bond	4.35	0.828
A valuable opportunity for the nursery to respond to mothers’ needs	4.35	0.795
A valuable opportunity for the nursery to respond to children’s needs	4.43	0.752
A way to promote greater parental involvement in the life of the nursery	3.71	1.061
A way to better understand mothers and children	3.84	1.045
An additional burden for educators	1.77	1.082
Something that may create difficulties for children who are not breastfed	1.58	0.845
Something that may create rivalry or distance among mothers who do not breastfeed their children	1.28	0.540
Something that interferes with the regular daily routine of the nursery	1.34	0.742

## Data Availability

The data presented in this study are available upon request from the corresponding authors. The data are not publicly available due to privacy.
